# Knowledge as a Predictor for Preparedness in Managing COVID-19 Among General Practitioners in Malaysia

**DOI:** 10.7759/cureus.63147

**Published:** 2024-06-25

**Authors:** Khasnur Abd Malek, Farnaza Ariffin, Sri Wahyu Taher, Noor Azah Abd Aziz, Boon-How Chew, Ping Foo Wong, Sazlina Shariff Ghazali, Adina Abdullah, Azah Abdul Samad, Ziti Akthar Sufian, Yung Wen Han, Wei Jie Lai, Christine Shamala Selvaraj

**Affiliations:** 1 Primary Care Medicine Department, Faculty of Medicine, Universiti Teknologi MARA (UiTM), Shah Alam, MYS; 2 Family Medicine, Klinik Kesihatan Simpang Kuala, Ministry of Health Malaysia, Alor Setar, MYS; 3 Department of Family Medicine, Universiti Kebangsaan Malaysia Medical Centre, Kuala Lumpur, MYS; 4 Family Medicine, Faculty of Medicine and Health Sciences, Family Medicine Specialists Clinic, Universiti Putra Malaysia, Serdang, MYS; 5 Family Medicine, Hospital Sultan Abdul Aziz Shah (HSAAS) Teaching Hospital, Family Medicine Specialists Clinic, Serdang, MYS; 6 Family Medicine, Klinik Kesihatan Cheras Baru, Ministry of Health Malaysia, Kuala Lumpur, MYS; 7 Department of Family Medicine, Faculty of Medicine and Health Sciences, Universiti Putra Malaysia, Serdang, MYS; 8 Department of Primary Care, University of Malaya Medical Center, University of Malaya, Petaling Jaya, MYS; 9 Family Medicine, Shah Alam Health Clinic, Ministry of Health Malaysia, Selangor, MYS; 10 Family Medicine, Klinik Kesihatan Seri Kembangan, Ministry of Health Malaysia, Selangor, MYS; 11 Family Medicine, EcoSoul Clinic, Shah Alam, MYS; 12 Family Medicine, Drs. Tong, Leow, Chiam & Partners, Kuala Lumpur, MYS; 13 Department of Primary Care Medicine, Faculty of Medicine, Universiti Malaya, Kuala Lumpur, MYS

**Keywords:** general practitioner, preparedness, knowledge, sars-cov-2, covid-19

## Abstract

Introduction

The COVID-19 pandemic has changed the working environment for general practitioners (GPs). GPs had to adapt quickly when care mitigation for mild COVID-19 in the community began. We assessed Malaysian GPs’ knowledge and preparedness to manage COVID-19.

Method

A cross-sectional online survey was conducted between May and October 2022 among the GPs. Emails were sent to GPs affiliated with the main GP organizations in Malaysia, such as the Academy of Family Physicians of Malaysia (AFPM). Additionally, participation was sought through social media groups, including the Association of Malaysian Islamic Doctors, the Federation of Private Medical Practitioners’ Associations Malaysia, and the Primary Care Network. Data was collected using a self-administered questionnaire on items related to knowledge and preparedness to manage COVID-19. The content was validated by six experts. Multiple logistic regression was used to determine the predictors for preparedness.

Results

A total of 178 GPs participated in this study. The mean age of the GPs was 41.8 (SD 12.37) years, 54.5% were males, 47.8% had a postgraduate qualification, and 68% had up to 10 years of general practice experience. Their practices are commonly solo (55.1%), located within an urban area (56.2%) and 47.2% operate 7 days a week. A majority of GPs (n = 124, 69.7%) had a good level of knowledge of COVID-19. In contrast, about a third (n = 60, 33.7%) had a good level of preparedness to manage COVID-19. GPs with a good level of knowledge of COVID-19 had 1.96 times the odds of having a good level of preparedness as compared to GPs with lower knowledge (OR = 2.11 (95% CI: 1.06, 4.18, *p* = 0.03)).

Conclusion

A good level of knowledge is a predictor for preparedness to manage COVID-19. Relevant and targeted measures to enhance knowledge for better preparedness among the GPs to respond to future pandemics are needed.

## Introduction

The World Health Organization (WHO) declared a global public health emergency on January 30, 2020, in response to the coronavirus disease 2019 (COVID-19) pandemic that originated in Wuhan, China [1]. COVID-19 has introduced countless challenges to global health systems forcing institutions and stakeholders to rapidly adapt to control the spread [2]. Following vaccination, most infected individuals develop mild symptoms and make a full recovery without needing hospital treatment but individuals with morbidities risk developing severe COVID-19 infection [1].

As the number of mild COVID-19 cases grew, Malaysia mitigated the care of mild COVID-19 in the community and implemented an array of measures across federal, state, and territory governments [3-5]. Asymptomatic and mildly symptomatic patients were advised for community monitoring, to decongest the hospital’s occupancy and burden, but on the other hand, had significantly increased the workload demand in the primary care setting. Comprehensive guidelines were shared with primary healthcare providers, including private general practitioners (GPs) [3]. The Greater Klang Valley Special Task Force (GKVSTF) is one of many initiatives made to enhance and support community monitoring and management. The measures included deploying healthcare personnel strategically, offering specialized training for remote and virtual home monitoring, and promoting collaboration between the public and private sectors, as well as non-governmental organizations (NGOs) [4,5]. With this new care management protocol involving private GPs, preparedness is an important aspect that needs to be enhanced. Studies have found several protective factors for preparedness among GPs to manage COVID-19, including receiving adequate information to manage COVID-19, and adequate personal protective measures, support, and training [6-8].

While the necessity of sufficient knowledge had been recognized for perceived preparedness during the early pandemic time [7-8], there are limited studies on knowledge and preparedness during the mitigation phase of care of patients with mild COVID-19. Knowledge represents an essential tool for health authorities to strengthen preparedness among healthcare providers during the pandemic [9]. Knowledge and preparedness at this mitigation phase are particularly relevant as they enable health authorities to adapt and specifically target their actions. Therefore, in this study, we aim to determine the association between knowledge and preparedness among Malaysian GPs to manage COVID-19.

## Materials and methods

Study design and participants

An online cross-sectional questionnaire survey was conducted between May 2022 and October 2022 during the commencement of the endemic phase in Malaysia. GPs were identified via professional groups and academic institutions. The online survey was distributed via email and social media, particularly Facebook and WhatsApp. The invitation letter included a brief description of the study and a URL link to the survey. In this study, a GP is defined as a doctor who practices in a private GP’s setting, in a full-time practice or full-time locum with a minimum of six months of experience. Exclusion criteria were participants working in public or university primary care settings.

Measurements

The overall survey proforma comprised 43 questions. Demographic characteristics (13 items) assessed by the questionnaire included age, gender, qualifications, years of GP experience, and information related to the clinic including the location, opening days, and operating hours. The other questionnaire parts are described as follows. Knowledge measures the practical knowledge of COVID-19 and consists of three main parts. Part one focuses on the nature and transmission of SARS-CoV-2 infection with eight items. Part two addresses dealing with suspected, probable, and confirmed COVID-19 cases, encompassing six items. Part three covers mild COVID-19 treatment and home monitoring, comprising three and seven items, respectively. These 24 items were designed based on careful considerations of SARS-CoV-2 information from the WHO [10] and home monitoring guidelines [11,12]. Each item is scored as ‘True’ (1), ‘False’ (0), or ‘Not sure’ (0), with a maximum knowledge score of 24. A good level of knowledge is defined when the GPs’ knowledge total score is at or more than 23, representing the 75th percentile of the total knowledge score [13].

Preparedness measures the perception of preparedness to manage COVID-19. The six-item questions were designed by adapting findings from previous studies examining healthcare workers’ viewpoints related to COVID-19 preparedness [14-18] and input from five GPs in Malaysia. These GPs provide care for mild cases at home, potentially facing challenges in resources and information. Preparedness encompasses training sufficiency, access to the latest COVID-19 guidelines and policy, staff’s ability to manage COVID-19 cases, availability of isolation areas, access to personal protective equipment, and support from the health authority to manage COVID-19. Each item measure used a Likert Scale rating, from (1) ‘strongly disagree’, (2) ‘disagree’, (3) ‘neutral’, (4) ‘agree’, to (5) ‘strongly agree’. The maximum score for preparedness is 30 and the minimum score is 6. A good level of preparedness is defined when the GPs’ preparedness total score is at or more than 24, representing the 75th percentile of the total score of preparedness [13].

Six expert panels including three GPs specialists, two infectious diseases specialists, and one public health specialist validated the questionnaire content using the scale-level content validity index based on the average method (S-CVI/Ave) [19]. An acceptable CVI value for six experts is at least 0.83 [20]. The S-CVI/Ave scores across all items in all the domains were between 0.95 and 1.0, indicating an acceptable CVI [19]. The researchers edited all items with the expert’s comments. The final survey went through a face validation to check the extent of the appropriateness of the questionnaire to measure its purpose, among 10 private GPs before distribution. There were no major issues identified. Participants were able to understand and felt the questions effectively captured the topic under investigation.

Data collection

We utilized the email list provided by the Academy of Family Physicians of Malaysia (AFPM), a leading institution for professional development and postgraduate education for GPs. Membership in AFPM is open to all registered medical practitioners who are GPs. Study invitations were extended to all registered GPs affiliated with AFPM. Additionally, invitations were circulated among three major GP professional groups: the Association of Malaysian Islamic Doctors, Federation of Private Medical Practitioners Associations Malaysia, and Primary Care Network, through social media channels such as WhatsApp and Facebook. Furthermore, the study invitation QR code was distributed during three primary care training workshops held in Klang Valley, Malaysia.

Upon accessing the online survey, GPs who identified themselves as private practitioners were directed to participate in the study, while those affiliated with universities or public institutions received a notification indicating their ineligibility and exclusion from the study. The online survey included an information sheet and consent form on the initial page, and participants indicated their consent by clicking ‘I agree’ before proceeding with the survey. Reminder messages were dispatched at intervals of 2, 4, and 6 weeks following the initial invitations to encourage participation.

Sample size calculation and statistical analysis

The sample size was determined using the ‘Sample size for Proportion’ tool from OpenEpi Version 3.01. The calculation incorporated data from Yanti et al.’s study which found 43.1% of healthcare providers to have good knowledge and preparedness [21]. With a significance level of 0.05, the estimated sample size required for the study was 179. Once the study data collection was closed, raw data were downloaded from Google form, cleaned in Microsoft Excel, and analyzed in IBM SPSS 25. We used mean and standard deviations for continuous variables and frequencies and percentages for categorical variables. Variable with p <0.20 in the simple logistic regression was entered into the multiple logistic regression to determine predictors for a good level of preparedness.

## Results

A total of 178 completed questionnaires were collected and included in the final analysis. As shown in Table 1, most responses were from the northern and central regions (68.5%). Participants mean age was 41.8 (SD 12.37). Almost a quarter (68%) had 10 or more years of experience in general practice with about 47.8% having postgraduate qualifications. More than half of the participants run solo practices (55.1%). Over half of the practices were located in an urban setting which was within 20 km from the city center (56.2%) and open 7 days a week (47.2%). Commonly reported daily operating hours were ‘others’ (43.8%) followed by 12 hours (34.3%). Almost a quarter (69.7%) demonstrated a good level of knowledge related to SARS-CoV-2 (total score > 75th percentile). Just under a third of the participants, 60 (33.7%) had a good level of preparedness to manage COVID-19 (total score > 75th percentile).

**Table 1:** Participants’ sociodemographic factors, clinical factors, and knowledge, and preparedness levels to manage COVID-19 (N = 178) N: Total number of participants in the sample; n (%): values are presented as the total number of participants in a subgroup in the sample numbers (percentage). Mean±SD = Values are presented as mean ± standard deviation.

Variables	n (%)	Mean, (SD)
Age (n = 177)		41.8 (12.37)
Gender		
Male	97 (54.5)	
Female	81 (45.5)	
Qualification (n = 177)		
Postgraduate	85 (48)	
Bachelor	92 (52)	
Years in general practice (n = 177)		
Less or equal to 10 years	120 (68)	
More than 10 years	57 (32)	
Clinic location based on regions		
Northern and Central	122 (68.5)	
Eastern and Southern	38 (21.4)	
East Malaysia	18 (10.1)	
Clinic type		
Solo	98 (55.1)	
Group/partnership	80 (44.9)	
Clinic location		
City center	65 (36.5)	
Urban (within 20 km from the city center)	100 (56.2)	
Rural (more than 20 km from city center)	13 (7.3)	
Clinic opening days		
Less than 6 days/week	94 (52.8)	
7 days/week	84 (47.2)	
Clinic operating hours		
12 hours per day	61 (34.3)	
18 hours per day	19 (10.7)	
24 hours per day	20 (11.2)	
Others	78 (43.8)	
Good level of knowledge		
No (score <75th percentile)	124 (69.7)	
Yes (score ≥75th percentile)	54 (30.3)	
Good level of preparedness		
No (score <75th percentile)	118 (66.3)	
Yes (score ≥75th percentile)	60 (33.7)	

Knowledge to manage COVID-19

Table 2 lists the items related to SARS-CoV-2 knowledge. In relation to the characteristics and spread of SARS-CoV-2, over 90% of the participants provided accurate responses to the knowledge questions. Nevertheless, only 3.5% accurately responded to the statement that ‘SARS-CoV-2 is transmitted through the transfusion of infectious blood and needle stick injuries’.

**Table 2:** Frequency of correct responses to knowledge items related to SARS-CoV-2 (N = 178) T: True; F: False N = Total number of participants in the sample n (%) = Values are presented as the total number of participants in a subgroup in the sample numbers (percentage)

No	Domain: Nature and transmission of the disease (correct answer)	The correct answer, n (%)
K1	The incubation period of SARS-CoV-2 is 2 to 7 days but can be up to 14 days (T)	169 (94.9)
K2	Fever, tiredness, and loss of appetite are common symptoms of SARS-CoV-2 infection in the first 48 hours (T)	171 (96.1)
K3	Nausea or vomiting can be a presenting symptom of SARS-CoV-2 infection (T)	163 (91.6)
K4	Patients with SARS-CoV-2 infection who have advanced age, multiple comorbidities, and are not fully vaccinated are at increased risk of developing severe disease (T)	177 (99.4)
K5	The gold standard approach to diagnose SARS-CoV-2 infection is by sampling respiratory specimens for reverse transcription polymerase chain reaction (RT-PCR) (T)	172 (96.6)
K6	The SARS-CoV-2 is transmitted through direct contact with small droplets and particles that contain the virus, especially through cough or sneeze (T)	176 (98.8)
K7	SARS-CoV-2 is transmitted by transfusion of infectious blood and through needle stick injuries (F)	7 (3.9)
K8	SARS-CoV-2 can be eliminated with the use of at least 70% alcohol (T)	148 (83.1)
Domain: Actions in dealing with suspected, probable, and confirmed cases (correct answer)		
K9	A waiter who comes to your clinic with a sore throat and fever but does not have close contact with SARS-CoV-2 infection requires a test for SARS-CoV-2 virus (T)	157 (88.2)
K10	Patients who self-tested positive for SARS-CoV-2 infection should self-report in their MySejahtera (T)	177 (99.4)
K11	Suspected cases of SARS-CoV-2 infection after triage should be taken into a separate area with good ventilation (T)	177 (99.4)
K12	Patients with suspected SARS-CoV-2 infection should be given an N95 mask as soon as possible (F)	97 (54.5)
K13	The use of personal protective equipment is necessary during oropharyngeal or nasopharyngeal swab procedures (T)	178 (100)
K14	Patients with suspected COVID-19 who are on nasal-delivered oxygen potentially can cause aerosolization of SARS-CoV-2 virus (T)	133 (74.7)
Domain: Treatment (correct answer)		
K15	Antibiotic prophylaxis should be used in patients with SARS-CoV-2 infection (F)	10 (5.6)
K16	Anti-pyretic can be given to treat fever and pain in patients with SARS-CoV-2 infection (T)	177 (99.4)
K17	Anti-hypertensive drugs should routinely be stopped in patients with SARS-CoV-2 infection (F)	3 (1.7)
Domain: Home monitoring (correct answer)		
K18	Patients with mild SARS-CoV-2 infection with stable ischemic heart disease can be given home monitoring (T)	128 (71.9)
K19	SARS-CoV-2-infected patients on home monitoring should be advised to comply with mask-wearing if in the presence of others (T)	177 (99.4)
K20	An ideal carer for SARS-CoV-2-infected patients on home monitoring would be a young, healthy, and vaccinated person (T)	172 (96.6)
K21	SARS-CoV-2-infected patients with newly developed symptoms such as difficulty breathing and chest pain require a doctor’s assessment (T)	178 (100.0)
K22	Pulse oximetry can be used among SARS-CoV-2-infected patients on home monitoring to identify progression to severe disease (T)	176 (98.9)
K23	SARS-CoV-2 infected patients on home monitoring with a persistent fever of 39°C (for four days) require hospital admission (T)	132 (74.2)
K24	SARS-CoV-2-infected patients on home monitoring can only be discharged when at least seven (7) days have passed since symptom onset (F)	140 (78.7)

In the domain concerning suspected, probable, and confirmed cases, the majority of participants responded accurately (>90%). However, only 54.5% of participants provided the correct response to the statement ‘Patients with suspected SARS-CoV-2 should be provided with an N95 mask as soon as possible (False)’. In the treatment domain, two items yielded the lowest correct responses: ‘Antibiotic prophylaxis should be utilized in patients with SARS-CoV-2 infection (False) (5.6%)’ and ‘Anti-hypertensive drugs should be routinely discontinued in patients with SARS-CoV-2 infection (False) (1.7%)’. Regarding home monitoring, generally, over a quarter of participants were able to provide accurate responses.

The average score for total knowledge was 21.7 (SD 1.44), ranging from a minimum score of 15 to a maximum score of 24. Nearly a quarter (69.7%) exhibited a commendable level of knowledge concerning SARS-CoV-2 (score ≥ 75th percentile).

Preparedness to manage COVID-19

Table 3 shows the items on preparedness to manage COVID-19. The majority of participants reported having good access to personal protective equipment (84.4%). However, less than half disagreed or remained neutral regarding the items ‘receiving sufficient training to manage COVID-19 patients’ (42.1%) and ‘receiving adequate support from national/regional/local public health authorities to manage COVID-19’ (42.7%).

**Table 3:** Summary of responses to items related to preparedness to manage COVID-19 (N = 178) N = Total number of participants in the sample n (%) = Values are presented as the total number of participants in a subgroup in the sample numbers (percentage)

No	Items	Strongly disagree, n (%) (score 1)	Disagree, n (%) (score 2)	Neutral, n (%) (score 3)	Agree, n (%) (score 4)	Strongly agree, n (%) (score 5)
P1	I have received sufficient training to manage COVID-19 patients.	2 (1.1)	13 (7.3)	60 (33.7)	82 (46.1)	21 (11.8)
P2	Every clinical staff at my workplace has easy access to the latest COVID-19 management policy.	1 (0.6)	19 (10.7)	46 (25.8)	87 (48.9)	25 (14.0)
P3	All clinical staff in my practice place can manage COVID-19 patients.	4 (2.2)	36 (20.2)	62 (34.8)	57 (32)	19 (10.7)
P4	There is a designated isolation area for patients with suspected COVID-19 in my practice.	13 (7.3)	33 (18.5)	27 (15.2)	76 (42.7)	29 (16.3)
P5	There is adequate access to personal protective equipment (PPE) in my practice.	4 (2.2)	0	23 (12.9)	72 (40.4)	79 (44.4)
P6	Our practice receives adequate support from national/regional/local public health authorities to manage COVID-19.	5 (2.8)	22 (12.4)	49 (27.5)	77 (43.3)	25 (14.0)

The average score for total preparedness was 21.7 (SD 4.02), ranging from a minimum score of 8 to a maximum score of 30. Approximately a third of GPs (33.7%) demonstrated a commendable level of preparedness in managing COVID-19 (score ≥ 75th percentile).

Simple logistic regression and multiple logistic regression

Table 4 presents the results from simple logistic regression. Among the variables examined, four variables showed a p-value of <0.20: clinic type, clinic operation days, clinic operational hours of 24 hours, and level of knowledge. These variables were consequently included in the multiple logistic regression (MLogR). Table 5 presents the results from MLogR. The MLogR revealed that possessing a good level of knowledge increased the odds of having a good level of preparedness by 2.11 times. The model demonstrated a good fit (X2 = 12.279, df = 6, N = 178, p < 0.05), explaining 9.29% of the variance in the good level of preparedness (Nagelkerke R Square).

**Table 4:** Simple logistic regression on factors associated with a good level of preparedness (N = 178) 1 = Reference group N = Total number of participants in the sample Emboldened*: Statistical significance at p < 0.20

Variables	Simple logistic regression analysis			
	Beta	Standard error (df)	OR (95%CI)	p-value
Age in years (n = 177)	-0.01	0.01 (1)	0.99 (0.97, 1.02)	0.91
Gender				
Male			1	Ref
Female	0.03	0.32 (1)	1.03 (0.55, 1.92)	0.92
Qualification				
Postgraduate			1	Ref
Bachelor	-0.03	0.32 (1)	0.97 (0.52, 1.80)	0.91
Years in general practice				
Less or equal to 10 years			1	Ref
More than 10 years	-0.20	0.34 (1)	1.23 (0.63, 2.37)	0.54
Clinic location based on regions				
Northern and Central			1	Ref
Eastern and Southern	-0.42	0.41 (1)	0.66 (0.29, 1.48)	0.31
West Malaysia	0.16	0.52 (1)	1.17 (0.42, 3.23)	0.76
Clinic type				
Solo			1	Ref
Group/partnership	0.51	0.32 (1)	1.67 (8.91, 3.12)	0.11*
Clinic location				
City center			1	Ref
Urban (within 20 km from the city center)	-2.01	0.65 (1)	0.81 (0.22, 2.93)	0.75
Rural (more than 20 km from city center)	-0.11	0.33 (1)	0.90 (0.47, 1.74)	0.75
Clinic opening days				
Less than 6 days/week			1	Ref
7 days/week	0.47	0.32 (1)	1.61 (0.89, 3.00)	0.14*
Clinic operating hours				
12 hours per day			1	Ref
18 hours per day	0.47	0.54 (1)	1.61 (0.56, 4.64)	0.38
24 hours per day	0.99	0.53 (1)	2.70 (0.96, 6.60)	0.06*
Others	-0.14	0.37 (1)	0.87 (0.42, 1,81)	0.71
Good level of knowledge				
No			1	Ref
Yes	0.67	0.34 (1)	1.96 (1.01, 3.79)	0.05*

**Table 5:** Multiple logistic regression on factors associated with a good level of preparedness (n = 178) 1 = Reference group Emboldened**: Statistical significance at p <0.05 Model assumptions were met.

Variables	Multiple logistic regression analysis			
	Adjusted beta	Adjusted standard error (df)	Adjusted OR (95%CI)	p-value
Clinic type				
Solo			1	Ref
Group/partnership	0.41	0.33 (1)	1.51 (0.79, 2.89)	0.21
Clinic opening days				
Less than 6 days/week			1	Ref
7 days/week	0.21	0.67 (1)	1.23 (0.60, 2.89)	0.58
Clinic operating hours				
12 hours per day			1	Ref
18 hours per day	0.39	0.58 (1)	1.48 (0.47, 4.63)	0.50
24 hours per day	0.88	0.57 (1)	2.42 (0.79, 7.38)	0.12
Others	-0.17	0.38 (1)	0.84 (0.40, 1.78)	0.66
Good level of knowledge				
No			1	Ref
Yes	0.74	0.35 (1)	2.11 (1.06, 4.18)	0.03**

## Discussion

Preparedness is crucial for effective primary care during the pandemic, particularly as GPs play a vital role in providing patient-centered care for mild COVID-19 cases in the community. The good level of preparedness among our study population was 33.7%, slightly exceeding the 26.1% reported among Australian GPs in December 2019 at the pandemic’s onset. The Australian study observed a notable improvement to 75.7% in preparedness 10 months into the pandemic [14]. Our observed percentage is higher than Stöcker et al. (2021) [6]. Their study was conducted among German GPs during the peak of the first COVID-19 wave in March-April 2020, where preparedness was only 8.84%. Our study was conducted during the implementation of community home monitoring for asymptomatic and mild symptomatic COVID-19 patients. These were supported by home monitoring guidelines [3] and the GKVSTF [5], focusing on enhancing the healthcare service system through a remote monitoring initiative for home care in central regions. Essential support for primary care providers is crucial for the effective implementation of interventions in community care delivery [22]. In the context of home monitoring interventions, Fulop et al. (2023) reported positive staff feedback in England for remote monitoring services, despite challenges such as low patient updates and incomplete data [23]. In Malaysia, significant investments in remote monitoring have been made through various mobile apps like MySejahtera, SELangkah, and PgCare [24]. Study participants may have benefited from policy and remote monitoring support, possibly contributing to the slightly elevated preparedness. Additional local data is needed to validate this observation.

Guerrisi et al. (2022) demonstrated higher COVID-19 preparedness levels (40.6% in France and 60.2% in Spain). This may be attributed to unique preparedness factors among their primary care doctors, including having a reduced sense of risk, sufficient information, and confidence in health authorities’ ability to enforce effective measures. [7] In addition, Tse et al. (2020) found high COVID-19 preparedness (86.2%) among primary care doctors in China, which was attributed to adequate training and support, leading to confidence in diagnosis and management [8]. Moving forward, it is crucial to capitalize on these positive aspects by promoting ongoing training, support, and collaboration with health authorities. This approach can enhance overall preparedness and confidence, ensuring a robust response to COVID-19 challenges.

In our study, only 42.1% of GPs felt adequately trained to manage COVID-19 and 42.7% perceived sufficient support from public health authorities. This is despite the availability of guidelines and the GKVSTF team's presence to assist GPs in remote and virtual home monitoring during the study period. Perceptions of inadequate training and insufficient support are widespread issues among GPs, as identified in various studies [25-28]. In line with our results, Pilbeam et al. (2022) reported that GPs in the United Kingdom felt they received inadequate guidance and lacked of support for their practices. Small practices’ structural limitations hindering adherence to guidelines were a primary concern, with no alternative measures suggested to address these challenges [25]. Sandberg et al. (2023) found almost similar challenges among GPs in Sweden, reporting a lack of resources from health authorities for GPs to continue delivering care to elderly patients in the community during COVID-19 [26]. Larkins et al. (2022) reported a deficiency in early planning for GPs in Australia, where the initial focus on preventing SARS-CoV-2 infection did not align with GPs’ needs for information on providing care for COVID-19 patients [27]. This mismatch was reported due to the minimal involvement of primary care providers in the early planning stages.

In terms of the factors associated with preparedness to manage COVID-19, this study found that a good level of knowledge was significantly associated with a good level of preparedness. There are no predictive analysis findings on knowledge and preparedness available for comparison. However, the finding was comparable to a study conducted in Europe, which found that the GPs who were highly informed (received a high level of information) felt more prepared to manage COVID-19 [7]. The study attributed the findings to the frequent contact and information delivery from health authorities to primary care physicians. Comparatively, in Australia, a study among GPs revealed a high level of preparedness and good knowledge of COVID-19 in the descriptive analysis. Despite this, their study found that GPs struggled to stay updated with regards to the COVID-19 treatment domain, and attributed it to the multiple sources of information received at a rapidly changing pace. This finding is similar to our findings, where participants had a lower percentage of correct answers for treatment domains, specifically on ‘antibiotic prophylaxis use’ (5.6%) and ‘withholding anti-hypertensive drugs’ (1.7%). Healthcare providers depend heavily on up-to-date information and guidelines to make effective decisions. Staying informed and current about rapidly changing COVID-19 information is vital for preparedness [29]. To complement GPs’ efforts to keep up-to-date on COVID-19 matters, direct communication between the health authorities and GPs should be conducted regularly, to facilitate the delivery of fast-changing information in a meaningful and contextual way.

Our study discovered years in GP training were not a significant factor in preparedness to manage COVID-19. Previous studies found mixed findings. A study by Adane et al. (2021) conducted among hospital-based doctors in Ethiopia did not find training years as a significant factor in preparedness [30]. In contrast, Chanie et al. (2020) found the working experience of fewer than five years to be a significant factor in the low level of preparedness among the frontline healthcare providers in a hospital-based center [31]. In our study, 68% had more than 10 years of experience in general practice. An explanation for these mixed findings could be attributed to different attributes within the population sample, which may have led to outcomes that differed from what was anticipated. It could also be that there were other confounding factors not controlled for in the study.

The GP clinic type was also not a significant factor in our study. More than half of our study participants (55.1%) worked in a solo practice. While no direct inferential study has explored the relationship between preparedness and the type of practice, Makowski et al. (2023) conducted a qualitative study among a majority of solo-practice German GPs (64%), and GPs identified key strategies for preparedness, including facilitating easy access to accurate information, providing centralized office information, enhancing collegial exchange and communication, and fostering collaboration with the inpatient sector [32]. Due to the limited data availability of these variables and preparedness among GPs, there is a need to examine these variables further to enhance understanding of the topic.

Limitations

A large percentage of the study participants achieved a high total score in knowledge. This suggests that a high ceiling effect may have occurred that may hinder the accuracy of data interpretation. The researchers took several measures to detect the ceiling effect by performing a pilot study and readjusting questions to allow variation in responses. Future studies should relook at the questionnaire items to minimize the ceiling effect. This cross-sectional design with a generalized sampling frame and non-probability sampling may introduce biases. Furthermore, we were not able to gather information about response rates. Therefore, we will need to interpret the study findings with caution. While there was a high number of patients on home monitoring for mild COVID-19 in Malaysia during the survey period, there is a possibility that recall bias occurred. The study conducted a face validation among 10 primary care specialists, and their feedback was considered. Subsequently, the questionnaire was revised to ensure clarity and precision in the questions. Nevertheless, this is a preliminary study within the local setting, and the findings were able to shed some understanding of the knowledge and preparedness among the GPs.

## Conclusions

Our study reveals that a good level of knowledge predicts preparedness among GPs in managing COVID-19. Tailored strategies addressing the unique needs of GPs are crucial in this context. For accurate and timely delivery of COVID-19 information, direct communication between health authorities and GPs is vital. Training should be focused on important aspects of COVID-19 treatment management that are dynamic, with supplementary updates with new pieces of information given systematically. Guidelines should also take into account the distinctive needs and limitations of GPs. This can be achieved by involving GPs as key stakeholders from the very beginning. GPs have a valuable role as they can input on the unique challenges faced by smaller practices and can impart practical solutions for tailored strategies to suit the varying capacities of GP practices in overcoming constraints within the GPs’ practice capacity.

These findings shed light on the challenges GPs faced during the pandemic, thus guiding future pandemic readiness by emphasizing key strategies for all practitioners, especially those in solo practices. By understanding and promoting factors such as collaboration, communication, and centralized information access identified in the study, healthcare systems can better equip GPs to respond effectively to future outbreaks.

## Appendices

**Table 6:** Study questionnaire

No	Characteristics	Items
	A1: Gender	□ Male □ Female
	A2: Age	_________________ years old
	A4: Your highest education level	□ Postgraduate in family medicine □ Postgraduate other than family medicine □ Diploma related to family medicine □Diploma other than family medicine □ Bachelor degree
	A5: Your experience as a general practitioner	________________ years
	A6: What type of GP practice are you working at?	□ Solo practice □ Group practice
	A7: Which state is your clinic located in?	□Perlis □Kedah □ Perak □ Pahang □ Kelantan □ Terengganu □Putrajaya □Selangor □Kuala Lumpur □ Negeri Sembilan □ Melaka □ Johor □ Sarawak □ Sabah □Labuan
	A8: Where is your clinic located?	□ City center □ Urban ( within 20 km from the city center) □ Rural ( > 20 km from the city center)
	A9: How many days a week does your clinic operate?	□1 day □2 days □3 days □4 days □5 days □6 days □7 days
	A10: What are your clinic opening hours?	□12 hours □18 hours □24 hours □others:

	Domain 1: Nature and transmission of the disease	True	False	Not sure
K1	The incubation period of SARS-CoV-2 is 2 to 7 days but can be up to 14 days (T)			
K2	Fever, tiredness, and loss of appetite are common symptoms of SARS-CoV-2 infection in the first 48 hours (T)			
K3	Nausea or vomiting can be a presenting symptom of SARS-CoV-2 infection (T)			
K4	Patients with SARS-CoV-2 infection who have advanced age, multiple comorbidities, and are not fully vaccinated are at increased risk of developing severe disease (T)			
K5	The gold standard approach to diagnose SARS-CoV-2 infection is by sampling respiratory specimens for reverse transcription polymerase chain reaction (RT-PCR) (T)			
K6	The SARS-CoV-2 is transmitted through direct contact with small droplets and particles that contain the virus, especially through cough or sneeze (T)			
K7	SARS-CoV-2 is transmitted by transfusion of infectious blood and through needle stick injuries (F)			
K8	SARS-CoV-2 can be eliminated with the use of at least 70% alcohol (T)			
2	Domain 2: Actions in dealing with suspected, probable, and confirmed cases			
K9	A waiter who comes to your clinic with a sore throat and fever but does not have close contact with SARS-CoV-2 infection requires a test for SARS-CoV-2 virus (T)			
K10	Patients who self-tested positive for SARS-CoV-2 infection should self-report in their MySejahtera (T)			
K11	Suspected cases of SARS-CoV-2 infection after triage should be taken into a separate area with good ventilation (T)			
K12	Patients with suspected SARS-CoV-2 infection should be given an N95 mask as soon as possible (F)			
K13	The use of personal protective equipment is necessary during oropharyngeal or nasopharyngeal swab procedures (T)			
K14	Patients with suspected COVID-19 who are on nasal-delivered oxygen potentially can cause aerosolization of SARS-CoV-2 virus (T)			
3	Domain 4: Treatment			
K15	Antibiotic prophylaxis should be used in patients with SARS-CoV-2 infection (F)			
K16	Anti-pyretic can be given to treat fever and pain in patients with SARS-CoV-2 infection (T)			
K17	Anti-hypertensive drugs should routinely be stopped in patients with SARS-CoV-2 infection (F)			
4	Dimension 5: Home monitoring			
K18	Patients with mild SARS-CoV-2 infection with stable ischemic heart disease can be given home monitoring (T)			
K19	SARS-CoV-2-infected patients on home monitoring should be advised to comply with mask-wearing if in the presence of others (T)			
K20	An ideal carer for SARS-CoV-2 infected patients on home monitoring would be a young, healthy, and vaccinated person (T)			
K21	SARS-CoV-2-infected patients with newly developed symptoms such as difficulty breathing and chest pain require a doctor’s assessment (T)			
K22	Pulse oximetry can be used among SARS-CoV-2 infected patients on home monitoring to identify progression to severe disease (T)			
K23	SARS-CoV-2-infected patients on home monitoring with persistent fever of 39°C (for four days) require hospital admission (T)			
K24	SARS-CoV-2-infected patients on home monitoring can only be discharged when at least seven (7) days have passed since symptom onset (F)			

No	Questions	Strongly disagree	Disagree	Neutral	Agree	Strongly agree
P1.	I have received sufficient training to manage COVID-19 patients					
P2	Every clinical staff at my workplace has easy access to the latest COVID-19 management policy					
P3	All clinical staff in my practice place can manage COVID-19 patients					
P4	There is a designated isolation area for patients with suspected COVID-19 in my practice					
P5	There are adequate access to personal protective equipment (PPE) in my practice					
P6	Our practice receives adequate support from national/ regional/ local public health authorities to manage COVID-19					
